# Chromosomal rearrangements played an important role in the speciation of rice rats of genus *Cerradomys* (Rodentia, Sigmodontinae, Oryzomyini)

**DOI:** 10.1038/s41598-023-50861-3

**Published:** 2024-01-04

**Authors:** Willam Oliveira da Silva, Stella Miranda Malcher, Malcolm Andrew Ferguson-Smith, Patricia Caroline Mary O’Brien, Rogério Vieira Rossi, Lena Geise, Julio Cesar Pieczarka, Cleusa Yoshiko Nagamachi

**Affiliations:** 1 Laboratório de Citogenética, Centro de Estudos Avançados da Biodiversidade, Instituto de Ciências Biológicas, Universidade Federal do Pará (UFPA), Belém, Pará Brazil; 2 Cambridge Resource Centre for Comparative Genomics, Department of Veterinary Medicine, University of Cambridge, Cambridge, UK; 3 Departamento de Biologia e Zoologia, Instituto de Biociências, Universidade Federal do Mato Grosso (UFMT), Mato Grosso, Brazil; 4 Departamento de Zoologia, Laboratório de Mastozoologia, Universidade do Estado do Rio de Janeiro, Rio de Janeiro, Brazil

**Keywords:** Evolution, Genetics

## Abstract

Rodents of the genus *Cerradomys* belong to tribe Oryzomyini, one of the most diverse and speciose groups in Sigmodontinae (Rodentia, Cricetidae). The speciation process in *Cerradomys* is associated with chromosomal rearrangements and biogeographic dynamics in South America during the Pleistocene era. As the morphological, molecular and karyotypic aspects of Myomorpha rodents do not evolve at the same rate, we strategically employed karyotypic characters for the construction of chromosomal phylogeny to investigate whether phylogenetic relationships using chromosomal data corroborate the radiation of *Cerradomys* taxa recovered by molecular phylogeny. Comparative chromosome painting using *Hylaeamys megacephalus* (HME) whole chromosome probes in *C. langguthi* (CLA)*, Cerradomys scotii* (CSC), *C. subflavus* (CSU) and *C. vivoi* (CVI) shows that karyotypic variability is due to 16 fusion events, 2 fission events, 10 pericentric inversions and 1 centromeric repositioning, plus amplification of constitutive heterochromatin in the short arms of the X chromosomes of CSC and CLA. The chromosomal phylogeny obtained by Maximum Parsimony analysis retrieved *Cerradomys* as a monophyletic group with 97% support (bootstrap), with CSC as the sister to the other species, followed by a ramification into two clades (69% of branch support), the first comprising CLA and the other branch including CVI and CSU. We integrated the chromosome painting analysis of Eumuroida rodents investigated by HME and *Mus musculus* (MMU) probes and identified several syntenic blocks shared among representatives of Cricetidae and Muridae. The *Cerradomys* genus underwent an extensive karyotypic evolutionary process, with multiple rearrangements that shaped extant karyotypes. The chromosomal phylogeny corroborates the phylogenetic relationships proposed by molecular analysis and indicates that karyotypic diversity is associated with species radiation. Three syntenic blocks were identified as part of the ancestral Eumuroida karyotype (AEK): MMU 7/19 (AEK 1), MMU 14 (AEK 10) and MMU 12 (AEK 11). Besides, MMU 5/10 (HME 18/2/24) and MMU 8/13 (HME 22/5/11) should be considered as signatures for Cricetidae, while MMU 5/9/14, 5/7/19, 5 and 8/17 for Sigmodontinae.

## Introduction

The employment of whole chromosome probes to study karyotype diversity provides precise information about the chromosomal rearrangements involved in intra- and interspecific differences among many species^1,2^, and identifies chromosomal traits that can be used as phylogenetic markers^3–6^. Indeed, the use of human whole chromosome probes (HSA) confirms the monophyly of Xenarthra (sloths, armadillos, and anteaters)^7^ and Afrotheria^8^. The detection of the trait HSA 2q/21 elucidates the ambiguous position of Dermoptera within Supraprimates and shows that Scandentia and Dermoptera are more phylogenetically related to each other than to Primates^9^; and in Phyllostomidae bats, the monophyly of the Phyllostomini tribe is confirmed by the use of *Carollia brevicauda* and *Phyllostomus hastatus* probes^10,11^.

The phylogenetic relationships of several Myomorpha rodents have been clarified by whole chromosome probes of *Mus musculus* (MMU; house mouse; Muridae)^12^, however MMU karyotypes are highly rearranged in comparison with Sigmodontinae and few syntenies are observed (e.g., MMU 3/18 and 6/12)^13^. Therefore, the use of whole chromosome probes from a Sigmodontinae taxon gives more accurate information of conserved syntenies and the phylogenetic relationships within this group. This has been observed in the Akodontini tribe (Sigmodontinae, Cricetidae), in which *Hylaeamys megacephalus* (HME) whole chromosome probes were used to identify chromosomal signatures that reinforce the weakly supported phylogenetic relationships within this tribe^6^. Further, Pereira et al.^14^ applied HME whole chromosome probes to *Necromys lasiurus* and *Akodon diauarum* (Sigmodontinae), a previously hybridized species using MMU probes^13^, and the results from both probe sets were linked together, allowing MMU syntenic blocks to be identified within Sigmodontinae species that were already hybridized with HME probes, such as MMU5/9, MMU5/7/19, MMU5/10, MMU3/18, MMU8/13 and MMU6/12^14^. In this work, Pereira et al.^14^ also identified syntenic blocks shared by representatives in both families (Cricetidae and Muridae), such as MMU5/6, MMU1/17, MMU10/17 and MMU 12/17, and are an indicative of part of the ancestral Eumuroida karyotype (AEK).

Chromosomal traits have been used to construct many phylogenies^11,15–21^. This strategy may be applied to explain divergence in Myomorpha rodents in which morphological, molecular and chromosomal traits did not evolve at the same rate as in the above groups^12,23^, resulting in different phylogenetic arrangements.

The genus *Cerradomys* (Rodentia, Sigmodontinae, Oryzomyini) consists of eight species, which exhibit a wide range of karyotypic diversity, with variation in diploid number (2n) from 46 in *C. langguthi* to 60 in *C. akroai*, and autosomal fundamental number (FNa) from 54 in *C. marinhus* to 76 in *C. akroai*, with variability in 2n and/or FNa within some lineages^24^. Recently, the genus *Cerradomys* (Rodentia, Sigmodontinae) has been investigated by a multidisciplinary approach (karyotypic, molecular and phylogeographic)^24,25^. In order to understand the chromosomal evolution of the group in the light of their molecular phylogeny, Di-Nizo et al.^24^ analysed karyotypic (classic cytogenetics and chromosome painting) and molecular (Cytochrome b—Cytb—and concatenated multi-locus; cyt-*b*, COI, IRBP and i7FBG)) data from eight species, with 2n 50 to 60 and FNa from 54 to 76^24,26^. These authors identified extensive genomic reshuffling as shown by chromosome painting with *Oligoryzomys moojeni* (Rodentia, Sigmodontinae) whole chromosome probes^27^, and used Cyt-b topology to shed light on chromosome evolution within this diverse group^24^. Indeed, a phylogeographic approach for the genus *Cerradomys* suggests that climate changes in the Pleistocene and other biogeographic changes that occurred in South America played an important role in diversification of this group, but chromosomal rearrangements may have facilitated the speciation processes^25^.

In view of the independent evolutionary pathways that chromosomal, morphological and molecular traits of Myomorpha rodents can follow, we set out to investigate if the phylogenetic relationships using chromosomal data corroborated the radiation of *Cerradomys* taxa recovered by the molecular phylogeny. To achieve this goal, we constructed a chromosomal phylogeny using data from chromosome banding and chromosome painting with HME whole chromosome probes^28^ in *Cerradomys scotii*, *C. subflavus* and *C. vivoi*, plus those applied in *C. langguthi* by Nagamachi et al.^28^. Here, we discuss karyotypic evolution within the genus, the role of chromosomal rearrangements in speciation, and compare our results with the other 21 Sigmodontinae taxa analyzed using the same set of probes^6,14,21,28–34^.

## Results

### Classic cytogenetics

**Figure 1:**
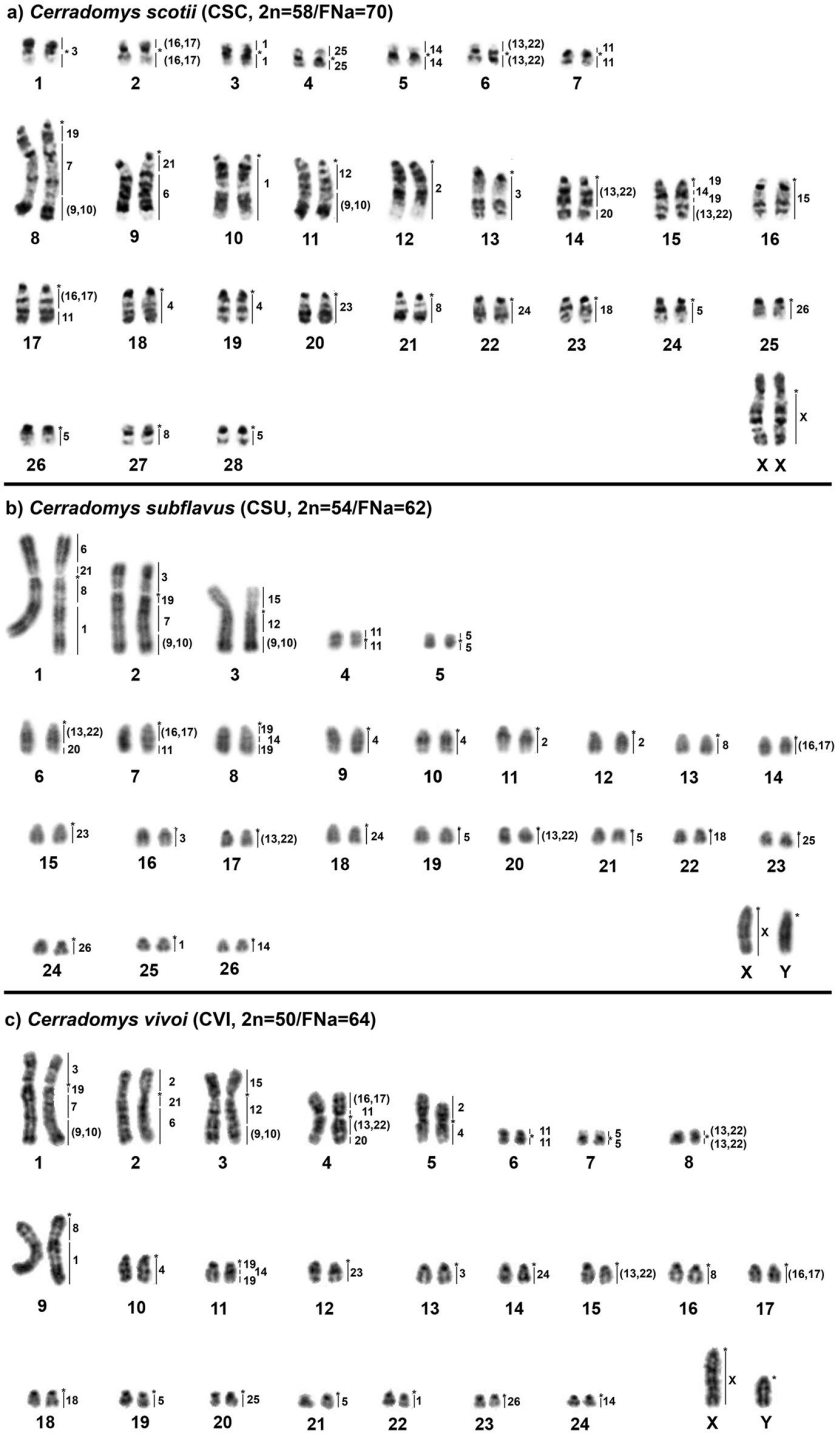
G-banded karyotypes with chromosome painting data revealed by *Hylaeamys megacephalus* (HME) whole chromosome probes^28^. (**a**) *Cerradomys scotii* (CSC), (**b**) *C. subflavus* (CSU) and (**c**) *C. vivoi* (CVI). An asterisk indicates a centromere.

**Figure 2:**
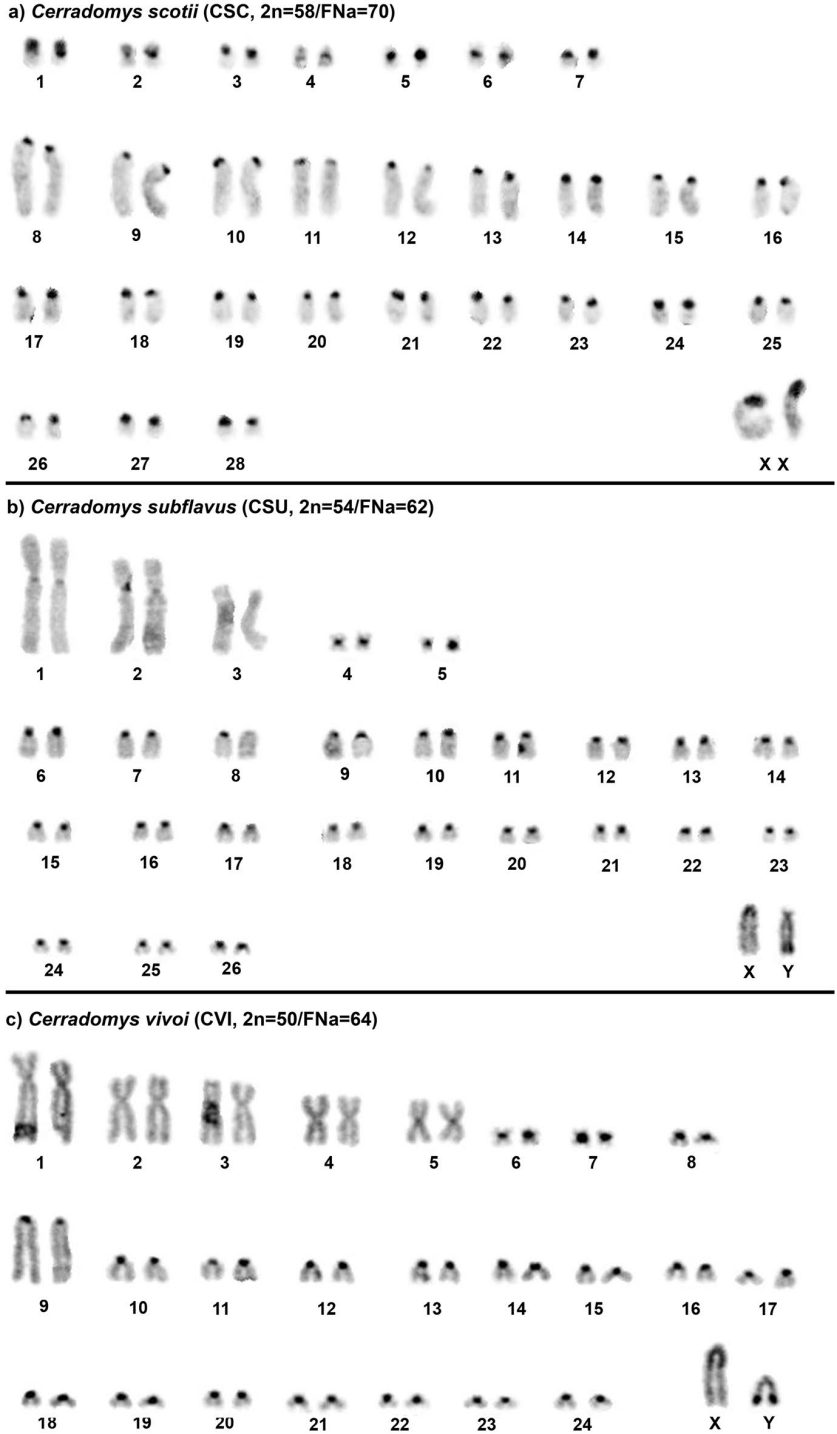
C-banded karyotypes of (**a**) *Cerradomys scotii* (CSC), (**b**) *C. subflavus* (CSU) and (**c**) *C. vivoi* (CVI).

*Cerradomys scotii* (CSC) has a 2n = 58/FNa = 70 karyotype; the autosomal set consists of 7 meta/submetacentric pairs (1–7) and 21 acrocentric pairs (8–28); the X chromosome is a large submetacentric (Fig. 1a); constitutive heterochromatin (CH) is distributed at the centromeric region of all autosomes; pairs 1, 3 (small submetacentrics) and the X chromosome exhibit large blocks of CH in their short arms (Fig. 2a). *Cerradomys subflavus* (CSU) has a 2n = 54/FNa = 62 karyotype; the autosomal set consists of 5 meta/submetacentric pairs (1–5) and 21 acrocentric pairs (6–26); the X and Y chromosomes are medium acrocentrics (Fig. 1b); CH is distributed at the centromeric region of all autosomes, except pair 3; the X chromosome exhibits CH at the centromeric region, and the Y chromosome exhibits CH at the distal region of the long arm (Fig. 2b). *Cerradomys vivoi* (CVI) has a 2n = 50/FNa = 64 karyotype; the autosomal set consists of 8 meta/submetacentric pairs (1–8) and 16 acrocentric pairs (9–24); the X chromosome is a large acrocentric, and the Y chromosomes is a medium acrocentric (Fig. 1c); the CH is distributed at the centromeric region of all autosomes, except pairs 1–4; the X chromosome exhibits CH at the proximal region, and the Y chromosome exhibits a block of CH at the distal region of the long arm (Fig. 2c).

### Molecular cytogenetics

Chromosome painting with all 24 *Hylaeamys megacephalus* (HME) whole-chromosome probes was performed on *Cerradomys scotii* (CSC), *Cerradomys subflavus* (CSU) and *Cerradomys vivoi* (CVI), and yielded 38, 39 and 39 hybridization signals, respectively.

**Figure 3:**
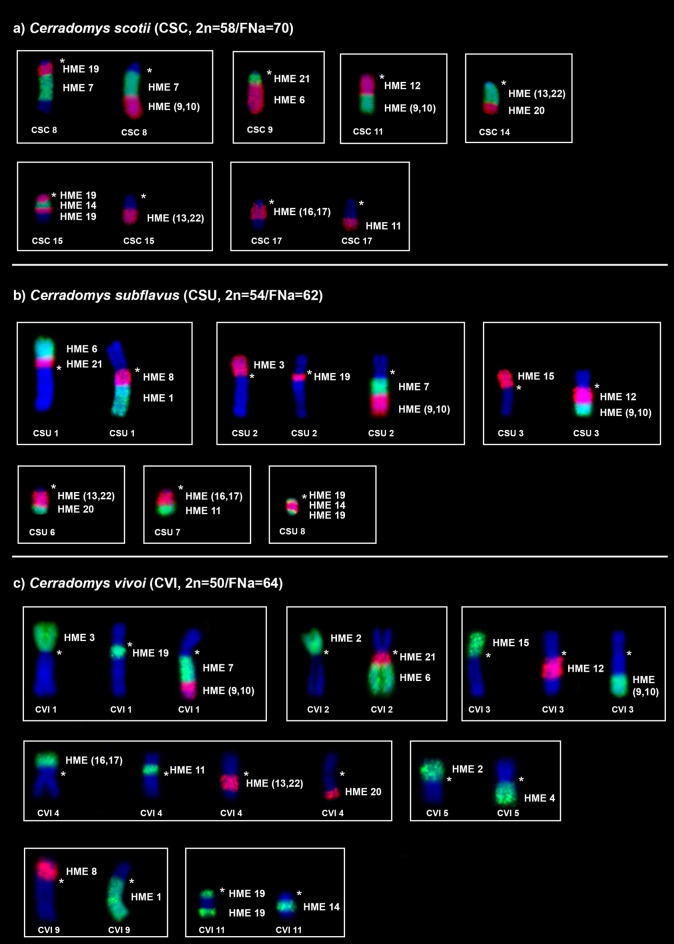
FISH results obtained from (**a**) *Cerradomys scotii* (CSC), (**b**) *C. subflavus* (CSU) and (**c**) *C. vivoi* (CVI). An asterisk indicates a centromere. Each box corresponds to a chromosomal pair that is composed of more than one HME homologue. Single or multiples images are addressed to exhibit full coverage with HME probes. HME whole chromosome probes are shown in green (FITC) and red (CY3); counterstaining is blue (DAPI).

HME probes yielded 38 hybridization signals in CSC chromosomes (Fig. 1a; Table 1). Twelve autosomal probes are conserved, of which seven (HME 2, 15, 18, 23, 24, 25 and 26) hybridized to whole-chromosomes of CSC (12, 16, 23, 20, 22, 4 and 25, respectively), and five (HME 6, 7, 12, 20, and 21) hybridized with portions of chromosomes of CSC (9q distal, 8q interstitial, 11q proximal, 14q distal and 9q proximal, respectively). Eleven autosomal probes showed multiple signals in CSC: HME 1 (CSC 3 and 10); HME 3 (CSC 1 and 13); HME 4 (CSC 18 and 19); HME 5 (CSC 24, 26 and 28); HME 8 (CSC 21 and 27); HME (9,10) (CSC 8q distal and 11q distal); HME 11 (CSC 7 and 17q distal); HME (13,22) (CSC 6, 14q proximal and 15q distal); HME 14 (CSC 5 and 15q interstitial); HME (16,17) (CSC 2 and 17q proximal); HME 19 (CSC 8q proximal, 15q proximal and interstitial); the HME X hybridized to CSC Xq, due to the presence of a heterochromatic block in the short arm. Six CSC autosomal pairs showed hybridization signals from multiple HME probes (Fig. 3a): CSC 8 (HME 19/7/(9,10)); CSC 9 (HME 21/6); CSC 11 (HME 12/(9,10)); CSC 14 (HME (13,22)/20); CSC 15 (HME 19/14/19/(13,22)); CSC 17 (HME (16,17)/11).

**Table 1:** FISH results for *Cerradomys* taxa as assessed based on hybridization with *Hylaeamys megacephalus* (HME) whole-chromosome probes^28^.

HME	CSC	CSU	CVI
1	3, 10	1q dist., 25	9q dist., 22
2	12	11, 12	2p, 5p
3	1, 13	2p, 16	1p, 13
4	18, 19	9, 10	5q, 10
5	24, 26, 28	5, 19, 21	7, 19, 21
6	9q dist	1p dist	2q dist
7	8q int	2q int	1q int
8	21, 27	1p prox., 13	9q prox., 16
(9,10)	8q dist., 11q dist	2q dist., 3q dist	1q dist., 3q dist
11	7, 17q dist	4, 7q dist	4p prox., 6
12	11q prox	3q prox	3q prox
(13,22)	6, 14q prox., 15q dist	6q prox., 17, 20	4q prox., 8, 15
14	5, 15q int	8q int., 26	11q int., 24
15	16	3p	3p
(16,17)	2, 17q prox	7q prox., 14	4p dist., 17
18	23	22	18
19	8q prox., 15q prox., 15q int	2q prox., 8q prox., 8q dist	1q prox., 11q prox., 11q dist
20	14q dist	6q dist	4q dist
21	9q prox	1p prox	2q prox
23	20	15	12
24	22	18	14
25	4	23	20
26	25	24	23
X	Xq	X	X

*Cerradomys scotii* (CSC; 2n = 58/FNa = 70); *C. subflavus* (CSU; 2n = 54/FNa = 62); *C. vivoi* (CVI; 2n = 50/FNa = 64).

HME probes yielded 39 hybridization signals in CSU chromosomes (Fig. 1b; Table 1). Eleven autosomal probes are conserved; of them, five (HME 18, 23, 24, 25 and 26) hybridized to whole chromosomes of CSU (22, 15, 18, 23 and 24, respectively) and six (HME 6, 7, 12, 15, 20 and 21) hybridized with portions of chromosomes of CSU (1p distal, 2q interstitial, 3q proximal, 3p, 6q distal and 1p proximal, respectively). Twelve autosomal probes showed multiple signals in CSU: HME 1 (CSU 1q distal and 25); HME 2 (CSU 11 and 12); HME 3 (CSU 2p and 16); HME 4 (CSU 9 and 10); HME 5 (CSU 5, 19 and 21); HME 8 (CSU 1p proximal and 13); HME (9,10) (CSU 2q distal and 3q distal); HME 11 (CSU 4 and 7q distal); HME (13,22) (CSU 6q proximal, 17 and 20); HME 14 (CSU 8q interstitial and 26); HME (16,17) (CSU 7q proximal and 14); HME 19 (CSU 2q proximal, 8q proximal and distal); HME X (CSU X). Six CSU autosomal pairs showed hybridization signals from multiple HME probes (Fig. 3b): CSU 1 (HME 6/21/8/1); CSU 2 (HME 3/19/7/(9,10)); CSU 3 (HME 15/12/(9,10)); CSU 6 (HME (13,22)/20); CSU 7 (HME (16,17)/11); CSU 8 (HME 19/14/19).

HME probes yielded 39 hybridization signals in CVI chromosomes (Fig. 1c; Table 1). Eleven autosomal probes are conserved, five of which (HME 18, 23, 24, 25 and 26) hybridized to whole chromosomes of CVI (18, 12, 14, 20 and 23, respectively), and six (HME 6, 7, 12, 15, 20 and 21) of which hybridized with portions of chromosomes of CVI (2q distal, 1q interstitial, 3q proximal, 3p, 4q distal and 2q proximal, respectively). Twelve autosomal probes showed multiple signals in CVI: HME 1 (CVI 9q distal and 22); HME 2 (CVI 2p and 5p); HME 3 (CVI 1p and 13); HME 4 (CVI 5q and 10); HME 5 (CVI 7, 19 and 21); HME 8 (CVI 9q proximal and 16); HME (9,10) (CVI 1q distal and 3q distal); HME 11 (CVI 4p proximal and 6); HME (13,22) (CVI 4q proximal, 8 and 15); HME 14 (CVI 11q interstitial and 24); HME (16,17) (CVI 4p distal and 17); HME 19 (CVI 1q proximal, 11q proximal and distal); HME X (CVI X). Seven CVI autosomal pairs showed hybridization signals from multiple HME probes (Fig. 3c): CVI 1 (HME 3/19/7/(9,10)); CVI 2 (HME 2/21/6); CVI 3 (HME 15/12/(9,10)); CVI 4 (HME (16,17)/11/(13,22)/20); CVI 5 (HME 2/4); CVI 9 (HME 8/1); CVI 11 (HME 19/14/19).

### Hybridization patterns among the three species of *Cerradomys*

From the 24 HME whole chromosome probes, five (HME 18, 23, 24, 25, 26) hybridized on whole chromosomes and five (HME 6, 7, 12, 20, 21) hybridized to part of one chromosome each on the three *Cerradomys* species; the HME 15 hybridized on a whole chromosome in CSC, and part of one chromosome in CSU and CVI; ten probes showed multiple signals, from them eight (HME 1, 3, 4, 8, (9,10), 11, 14, (16,17)) hybridized in distinct patterns among the three species: exhibiting signals only at whole chromosomes (CSC, CSU), signals in one whole chromosome and part of one chromosome (CSC, CSU, CVI); signals only at part of chromosomes (CSC, CSU, CVI); the remaining two probes (HME (13,22), 19) showed three signals, with HME (13,22) hybridizing from one (CSC) to two (CSU, CVI) chromosomes, and from part of one chromosome (CSU, CVI) to parts of two chromosomes (CSC); while HME 19 hybridized of parts of three chromosomes in each species.

The HME 2 was the only probe with difference in the number of signals, from one to two signals among *Cerradomys* species: hybridizing in one whole chromosome (CSC 12); in part of a chromosome and in a whole chromosome (CSU 1q distal, 25; CVI 9q distal, 22); the HME X chromosome hybridized to Xq due to the presence of a large heterochromatic block at CSC Xp.

According to chromosomal associations, the three taxa share the block HME 19/7/(9,10), but in CSU and CVI it is fused with HME 3 (HME 3/19/7/(9,10)); HME 6/21 is found in CSC, but it is fused with distinct blocks in CSU (HME 6/21/8/1) and CVI (HME 2/21/6). All three taxa share the HME 12/(9,10) block, but in CSU and CVI it is fused with HME 15 (15/12/(9,10)). In CSU and CSC, the blocks HME (13,22)/20 and (16,17)/11 are found as independent chromosomes, but in CVI, they are fused (HME (16,17)/11/(13,22)/20). Both CSU and CVI have HME 19/14/19 as independent chromosomes, but CSC has it fused with HME (13,22), resulting in HME 19/14/19/(13,22). Only CVI has the syntenic blocks HME 2/4 and 8/1.

### Regions of homology between MMU and HME

**Figure 4:**
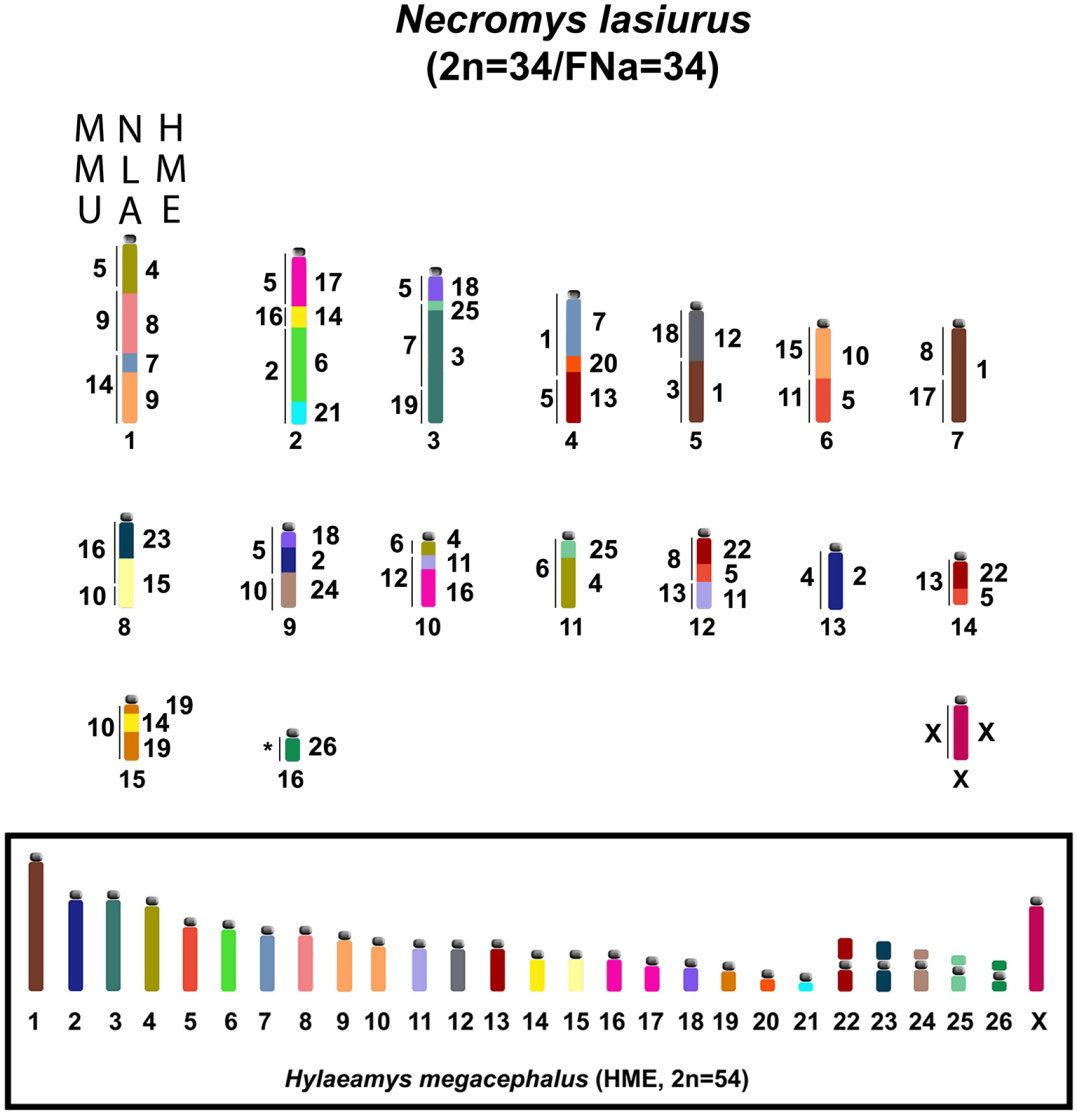
Idiogram of *Necromys lasiurus* (NLA)^14^. Homologies with HME (right) and MMU probes (left) are shown. The box contains an idiogram of the HME karyotype previously elaborated^21^ and adapted in Oliveira da Silva et al.^33^. Each HME chromosome is shown with a separate color, except the pairs (9,10), (13,22) and (16,17), which have one color each.

An analysis of 58 rodents hybridized by HME and MMU whole chromosome probes from the Cricetidae (Arvicolinae, Cricetinae, Neotomyinae, Sigmodontinae) and Muridae (Murinae, Deomyinae, Otomyinae) families was conducted (Additional File 2: Table S2). A homology was established between the two sets of probes using the karyotype of *Necromys lasiurus* (NLA, Sigmodontinae) investigated by HME^14^ and by MMU probes^13^. Accordingly, we created an idiogram of the NLA karyotype (Fig. 4)^14^, which allowed us to establish homology between the syntenic blocks of the 37 previously analyzed species and the 21 new species included in this study.

We identified 17 syntenic blocks (Table 2), from them the MMU 7/19 (HME 25/3) is found in representatives of the seven subfamilies from Cricetidae and Muridae; five syntenic blocks occurs in a variety of members of both families: the MMU 6/12 (HME 4/11/16) is absent only in Cricetinae and Neotomyinae; the MMU 14 (HME 7/9) is present in Sigmodontinae, Neotomyinae, Murinae and Otomyinae; the MMU 5/9 (HME 4/8) is found in Sigmodontinae, Cricetinae, Murinae and Deomyinae; the MMU 18/3 (HME 12/1) is found in Sigmodontinae and Deomyinae; MMU 12 (HME 11/16) is found in Sigmodontinae and Murinae. The remaining 10 syntenic blocks are found only in Cricetidae: MMU 5/10 (HME 18/2/24) is found in Sigmodontinae and Arvicolinae; MMU 8/13 (HME 22/5/11) is found in Sigmodontinae and Cricetinae; MMU 8 (HME 22/5) is found in Sigmodontinae and Cricetinae. The remaining seven syntenic blocks were found only in Sigmodontinae: MMU 5/9/14 (HME 4/8/7/9), MMU 5/7/19 (HME 18/25/3), MMU 5 (HME 18/2), MMU 8/17 (HME 1), HME 19/14/19, 20/13 and 26.

**Table 2:** Syntenic blocks shared among representatives of the Muroidea Superfamily were detected by whole chromosome probes of *Mus musculus* (MMU) and *Hylaeamys megacephalus* (HME).

Syntenic blocks			Cricetidae family				Muridae family		
HME	MMU	AEK	Sigmodontinae	Arvicolinae	Cricetinae	Neotomyinae	Murinae	Deomyinae	Otomyinae
4/8/7/9	5/9/14		+						
4/8	5/9		+		+		+	+	
18/25/3	5/7/19		+						
25/3	7/19	1	+	+	+	+	+	+	+
12/1	18/3		+					+	
18/2/24	5/10		+	+					
18/2	5		+						
4/11/16	6/12		+	+			+	+	+
11/16	12	11	+	+			+		
22/5/11	8/13		+			+			
22/5	8		+		+				
7/9	14	10	+			+	+		+
6/21	2		+		+	+	+		
1	8/17		+						
19/14/19			+						
20/13			+						
26			+						

Regions of homology between MMU and HME are established based on Fig. 4 and regions of homology between MMU and AEK (Ancestral Eumuroida karyotype) are established based on Romanenko et al.^35^. Presence of the syntenic block (+). See Additional File 2: Table S2 to access all taxa analyzed.

We highlight that based on the comparative analysis, three syntenic blocks were identified as part of the ancestral Eumuroida karyotype (AEK): MMU 7/19 (AEK 1), MMU 14 (AEK 10) and MMU 12 (AEK 11) (Table 2).

### Phylogenetic analysis

**Figure 5:**
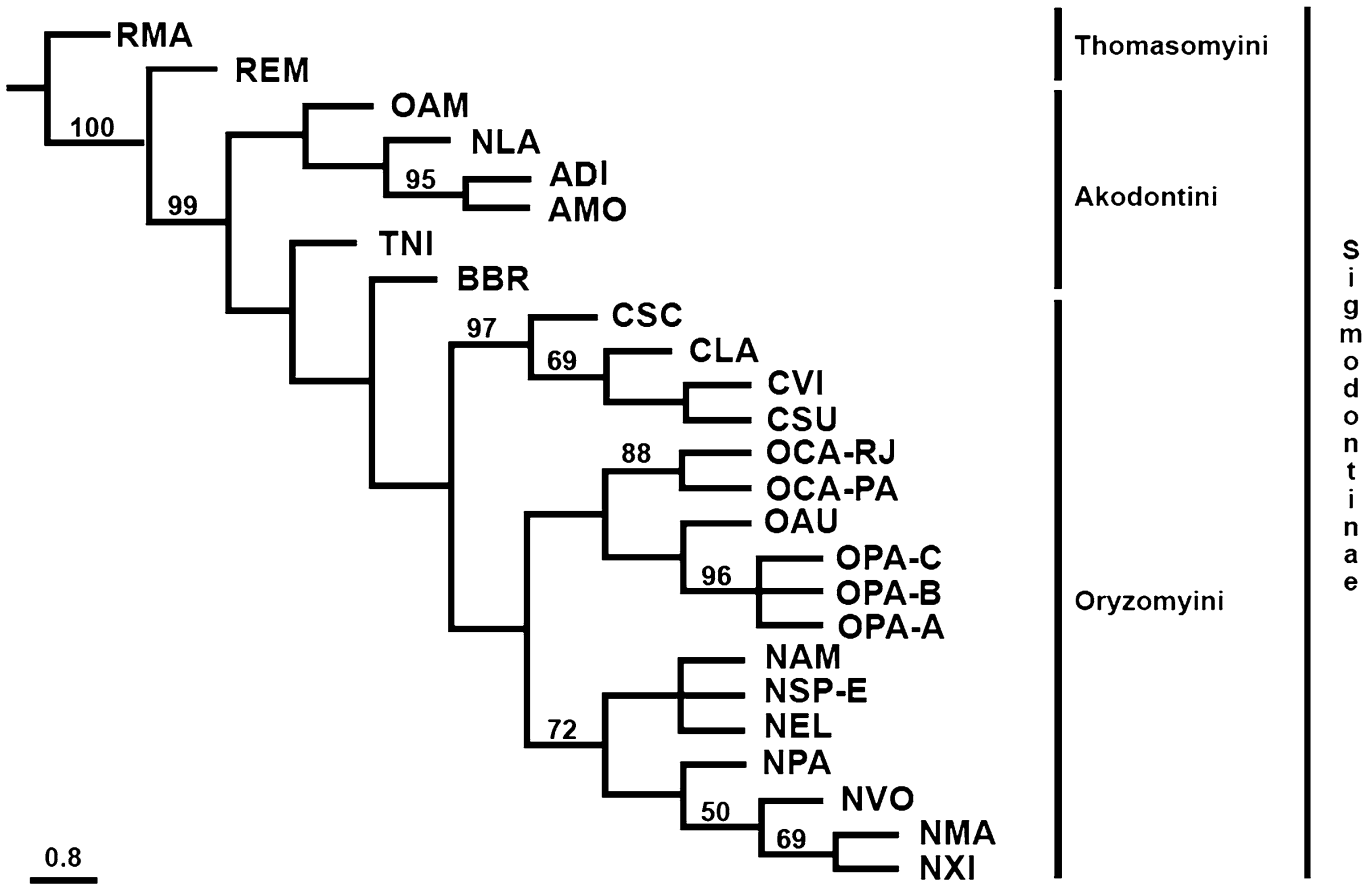
Most parsimonious tree based on matrix of chromosomal characters in Sigmodontinae taxa. Numbers above branches are Maximum Parsimony (MP) bootstrap values analysed on T.N.T. Only values of 50% and above are shown. A scale representing the distance among species based on accumulated character data is displayed.

The exhaustive analysis retrieved the most parsimonious tree and showed a best score of 136 evolutionary steps in a single tree from a total of 147,195 rearrangements. Bootstrap values ranged from 30 to 100% (Fig. 5). The Sigmodontinae subfamily showed an initial branching with *Rhipidomys mastacalis* (RMA), followed by *Rhipidomys emiliae* (REM) (100%), both representatives of Thomasomyini; the next ramification (99%) included *Oxymycterus amazonicus* (OAM), *Necromys lasiurus* (NLA), *Akodon diauarum* (ADI) and *Akodon montensis* (AMO) (95%), followed by two ramifications with *Thaptomys nigrita* (TNI) and *Blarinomys breviceps* (BBR) (Akodontini).

The next clade, composed only of Oryzomyini members recovered the genera *Cerradomys* as monophyletic (97%), with *C. scotii* (CSC) as sister to the other taxa, followed by *C. langguthi* (CLA) (69%), and the most derivate branch with *C. subflavus* (CSU) and *C. vivoi* (CVI). The other clade split into the genera *Oecomys* and *Neacomys*. The genus *Oecomys* grouped with high support values in terminal branches, grouping *O. catherinae* from Rio de Janeiro (OCA-RJ) and *O. catherinae* from Pará (OCA-PA) with 88% of support, followed by *O. auyantepui* and a polytomy with 96% that included *O. paricola* cytotypes A, B and C (OPA-A, OPA-B, OPA-C). The final clade is composed of *Neacomys* taxa with 72% of support, with the first ramification including a polytomy with *N. amoenus* (NAM), *Neacomys* sp. E (NSP-E), and *N. elieceri* (NEL). The next branch includes *N. paracou* (NPA), followed by *N. vossi* (50%) and the final ramification with *N. marajoara* (NMA) and *N. xingu* (NXI, 69%). It is noteworthy that since there is a low number of taxa and characters analysed, species exhibited high levels of autapomorphy, lowering the support values.

## Discussion

### Chromosomal rearrangements and signatures in *Cerradomys* (Rodentia, Sigmodontinae)

**Figure 6:**
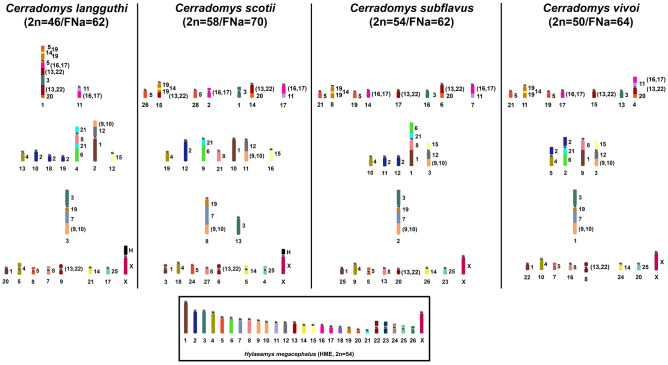
Idiograms of the karyotypes of *Cerradomys langguthi*^28^, *C. scotii*, *C. subflavus* and *C. vivoi* (present study). The karyotypic content of chromosomes involved in rearrangements of each species is separated into columns; the syntenic blocks involved in the rearrangements are arranged horizontally. The box contains an idiogram of HME karyotype previously elaborated^21^ and adapted in Oliveira da Silva et al.^33^. Each HME chromosome is shown with a separate color, except the pairs (9,10), (13,22) and (16,17), which have one color each. (H) indicates large block of constitutive heterochromatin.

Comparative chromosome painting analysis among *Cerradomys langguthi* (CLA) 2n = 46/FNa = 62^28^, *C. scotii* (CSC) 2n = 58/FNa = 70, *C. subflavus* (CSU) 2n = 54/FNa = 62, and *C. vivoi* (CVI) 2n = 50/FNa = 64 (present study) showed that the variability in 2n from 46 to 58 and FNa from 62 to 70 is due to 16 fusion events, 2 fission events, 10 pericentric inversions and 1 centromeric repositioning among autosomes of CLA (17 pairs), CSC (23 pairs), CSU (21 pairs) and CVI (19 pairs), plus two events of amplification of constitutive heterochromatin in the short arms of the X chromosomes of CLA and CSC (Fig. 6). Only five syntenic blocks were observed without detectable rearrangements, hybridized by HME 11, 18, 23, 24 and 26 probes. We also described an exclusive trait for this genus, the chromosomal association HME 19/7/(9,10).

We were unable to compare the chromosome painting data obtained in the present study with those previously published by Di-Nizo et al.^24^, since they did not use the entire set of *Oligoryzomys moojeni* (OMO) whole chromosome probes and, therefore, there were gaps in the karyotypes of *Cerradomys* taxa analyzed by them (*C. marinhus*, *C. maracajuensis*, *C. scotii*, *C. langguthi*, *C. vivoi*, *C. goytaca* and *C. subflavus*). Nevertheless, we corroborate the statement by Di-Nizo et al.^24^ that centric fusion/fission, centromere repositioning and pericentric inversions were responsible for the complex scenario that generated the karyotypes of *Cerradomys*. We did not detect any paracentric inversions and this is explained by the different sets of probes used by Di-Nizo et al.^24^ (OMO) and the present work (HME).

In light of the fact that the current karyotypes of HME^28^ and OMO^36^ are the result of independent rearrangements that occurred during the evolutionary process involving syntenic blocks that are shared between species, chromosome origins are also independent. Thus, probes corresponding to isolated chromosomes reflect this pattern as well. In this regard, the use of either HME or OMO whole chromosome probes as comparative reference sets can detect different arrangements of syntenic blocks within the target species. Accordingly, reciprocal chromosome painting revealed multiple translocations between *P. roberti* (PRO, 2n = 30) and *P. goeldii* (PGO, 2n = 25♂), responsible for the diversity between these species, with the conservation of only three chromosomes. In a multidirectional FISH analysis of *Proechimys* gr. *goeldii* (PGG, 2n = 16♀17♂), the use of PRO probes allowed to identify 16 fusion/fission events, five translocations, and two inversions, whereas the use of PGO probes allowed to detect 10 fusion/fission events and two inversions^37^. In this way, these data demonstrate the different arrangements of syntenic blocks that can be identified for the same species based on the probe set used.

In order to infer the chromosomal evolution of *Cerradomys* genus, karyotypic data was plotted on a Cyt-b phylogeny^24,25^ and on a concatenated phylogeny (Cyt-b, COI, IRBP and i7FBG)^25^, with *C. maracajuensis* (2n = 56,58/FNa = 58,60) and *C. marinhus* (2n = 56/FNa = 54) exhibiting the most conserved karyotypes while the remaining taxa underwent a complex karyotypic evolutionary process with a high rate of rearrangements^24^, with variation in 2n and FNa: *C. scotii* (2n = 58/FNa = 70–72), *C. akroai* (2n = 60/FNa = 74,76), *C. langguthi* (2n = 46,48,49,50/FNa = 56,62), *C. vivoi* (2n = 50/FNa = 62–64), *C. subflavus* (2n = 54-56FNa = 62–64), and *C. goytaca* (2n = 54/FNa = 62,63,66)^25,26,28,38,39^. In *C. maracajuensis*, predominates pericentric inversions as well as in *C. scotti*, with fusion/fission events in the latter one; *C. langguthi* and *C. vivoi* exhibited several fusion/fission events, while *C. goytaca* and *C. subflavus* exhibited fusion rearrangements and peri- paracentric inversions^24^. Therefore, the proposition that *Cerradomys* has distinct rates of chromosomal rearrangements^24^ is in agreement with our results, as we observed a high rate of chromosomal rearrangements that differentiate the karyotypes of CLA, CSC, CSU and CVI from each other. Similarly, in the speciose genus *Nannospalax* (Rodentia, Spalacidae), the evolutionary processes could not be fully understood^40^, for karyotype evolution was found to be “complicated and variable” with many parallel courses of speciation in this genus, as fusion events would be the evolutionary pattern for *N. leucodon* and some representatives of *N. xanthodon*, while fission events would apply to *N. ehrenbergi*.

### Chromosomal phylogeny

The use of whole chromosome probes for the detection of chromosomal signatures has elucidated the phylogenetic relationships of several mammalian lineages, such as Xenarthra^7^, Afrotheria^8^, Supraprimates^9^, and Chiroptera^10,11,18,20^. In relation to rodents, although the *Mus musculus* (Muridae) whole chromosome probes have been largely used in the chromosomal studies of Myomorpha rodents^12,21^, their application in Sigmodontinae (Cricetidae) taxa “rendered difficult interpretations”^13^. Instead, the application of HME whole chromosome probes has provided clear and concise data and has been valuable in solving phylogenetic questions in Sigmodontinae^6,14,21,28–34^. However, a homology established between the two sets of probes using the karyotype of *Necromys lasiurus* (NLA, Sigmodontinae) investigated by HME^14^ and by MMU probes^13^ suggested several syntenic blocks shared among taxa from families Muridae and Cricetidae.

The chromosomal evolution of Sigmodontinae taxa has been discussed previously based on HME probes, with the following chromosomal traits being proposed: for Sigmodontinae HME 1a, 1b, 7/(9,10), 8, 1/12, 6/21, 11/(16,17), 5/(16,17), 20/(13,22), 15, 19/14/19, 24, and 26; for Oryzomyini HME 8a, 8b, 18, and 25; for Akodontini HME 3/25; and for Thomasomyini HME (13,22)/26/25/3, 5/15^6,20,27,28,31,33^. We agree with those proposals, however the chromosomal trait HME 3/25 (MMU 7/19) proposed for Akodontini was observed in RMA and REM, both representatives of Thomasomyini^34^, which indicates phylogenetic proximity between these two tribes, as showed by Parada et al.^41^.

We agree that the block MMU 7/19 (AEK1) is conserved in Muroidea^14,35^, as it is present in all subfamilies investigated here (Table 2, Additional File 2: Table S2). We highlight that solely *Oligoryzomys flavescens* from the Oryzomyini tribe exhibits the MMU 7/19 (HME 3/25), while being absent in all other 17 Oryzomyini taxa investigated here.

Although the MMU 6/12 (HME 4/11/16) have been proposed as syntenic block shared between Sigmodontinae and Arvicolinae (Cricetidae)^14^, we identified this block in Murinae, Deomyinae and Otomyinae (Muridae), indicating that it is conserved in Muroidea; also, the dissociated form MMU 12 (HME 11/16) is present in Sigmodontinae, Arvicolinae, and Murinae (Muridae), which corresponds to the AEK 11^35^. Another syntenic block found conserved in both families Cricetidae (Sigmodontinae, Neotomyinae) and Muridae (Murinae, Otomyinae) is MMU 14 (HME 7/9), and corresponds to AEK 10. MMU 18/3 was previously described only in Sigmodontinae^14^, but we identified in *Acomys dimidiatus* (Deomyinae), being conserved in Muroidea. The MMU 5/10 (HME 18/2/24) and MMU 8/13 (HME 22/5/11) should be considered as signatures for Cricetidae, while MMU 5/9/14, 5/7/19, 5 and 8/17 for Sigmodontinae.

The molecular analysis using cyt-b and concatenated multi-locus (cyt-*b*, COI, IRBP and i7FBG) based on Bayesian Inference (BI) and Maximum Likelihood (ML) analysis^25^ recovered the genus *Cerradomys* as monophyletic, with *C. marinhus* + *C. maracajuensis* as sister to a large clade comprising the six remaining species analyzed; this latter clade splits into two major groups, the first containing *C. akroai* and *C. scotii* and the second retaining *C. langguthi* as sister to the following two subclades: *C. goytaca* + *C. subflavus*, and *C. vivoi*.

Although our chromosomal phylogeny has four (CLA, CSU, CSC and CVI; Fig. 5) out of the eight *Cerradomys* species recovered in the molecular analysis, our sample are included in two out of the three major clades recovered by Di-Nizo et al.^25^. By comparing both topologies, we observed that the chromosomal phylogeny recovered a similar reconstruction as the molecular phylogeny, with CSC as sister to the other taxa, followed by CLA, and the most derivate branch with CSU and CVI.

**Figure 7:**
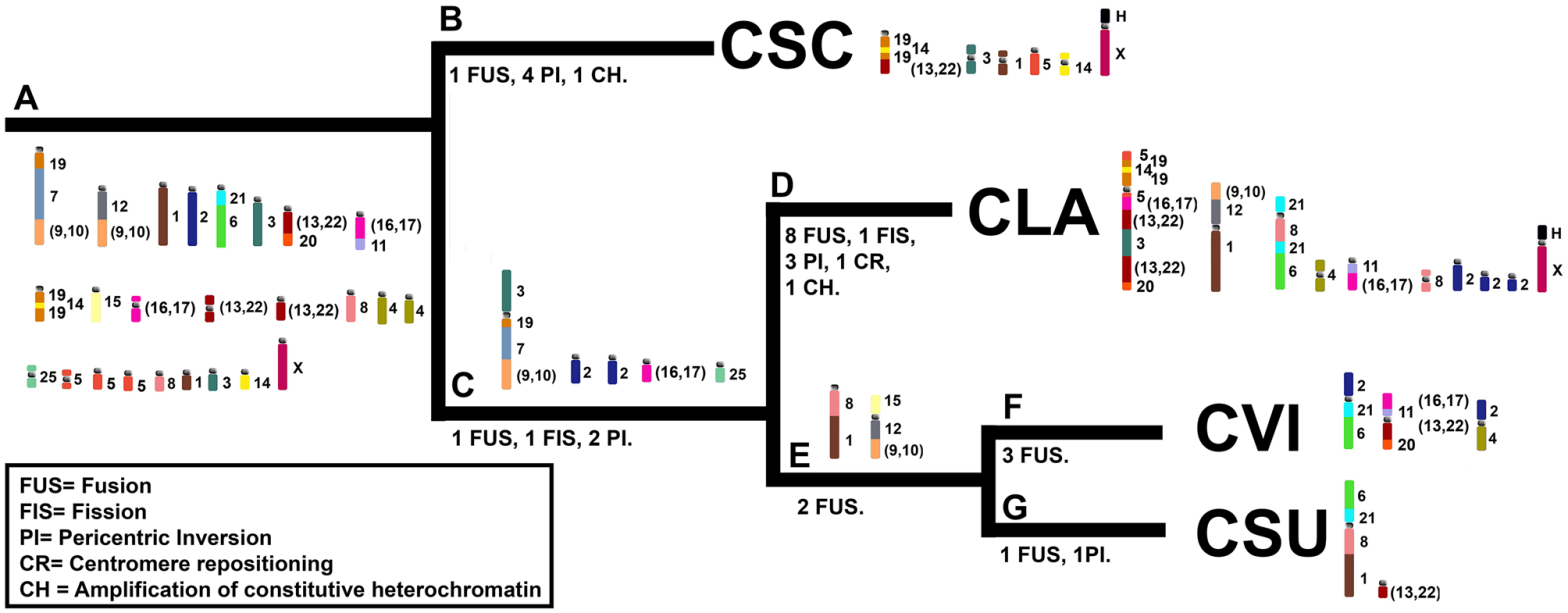
Direction of chromosomal rearrangements in *Cerradomys*. Fragment of the chromosomal phylogeny obtained in Fig. 5. Only idiograms of the chromosomes involved in rearrangements of *Cerradomys* taxa are displayed (Node A) and showing direction of chromosomal rearrangements (Nodes B-G) that shaped extant karyotypes of *C. scotii* (CSC), *C. langguthi* (CLA), *C. vivoi* (CVI) and *C. subflavus* (CSU). Idiograms are shown based on the HME karyotype (see Fig. 6). Each node indicates the number and type of chromosomal rearrangements that occurred during taxa diversification. The box indicates acronyms for chromosomal rearrangements and its respective legends.

We have identified 24 autosomal blocks involved in rearrangements: HME 19/7/(9,10), 12/(9,10), 1, 2, 21/6, 3, (13,22)/20, (16,17)/11, 19/14/19, 15, (16,17), (13,22), (13,22), 8, 4, 4, 25, 5, 5, 5, 8, 1, 3 and 14 (Node A, Fig. 7). In light of the chromosomal phylogeny, we propose the direction in which rearrangements may have occurred during speciation events in *Cerradomys* that shaped the extant karyotypes: as CSC is the first taxa to diverge (Node B, Fig. 7), we identified 1 fusion (HME 19/14/19/(13,22)) and four pericentric inversions (HME 3, 1, 5, 14); in the other clade (Node C, Fig. 7), 1 fusion (HME 3/19/7/(9,10)), 1 fission (HME 2a, 2b) and 2 pericentric inversions (HME (16,17), 25) occurred before the diversification of remaining taxa; in CLA (Node D, Fig. 7) we observed 8 fusions (HME 5/19/14/19/5/(16,17)/(13,22)/3/(13,22)/20, (9,10)/12/1, 21/8/21/6), 1 fission (HME 2a, 2b, 2c), 3 pericentric inversions (HME 21/8/21/6, 4, 8) and 1 centromere repositioning (HME 11/(16,17)); before the ramification of CVI and CSU (Node E, Fig. 7), two fusions occurred (HME 8/1, 15/12/(9,10)); in CVI (Node F, Fig. 7), we identified 3 fusions (HME 2/21/6, (16,17)/11/(13,22)/20, 2/4); in CSU (Node G, Fig. 7), we identified 1 fusion (HME 6/21/8/1) and 1 pericentric inversion (HME (13,22)). We also identified that CSC and CLA exhibit large blocks of constitutive heterochromatin in the short arms of the X chromosomes. Although CSC and CLA are the first to diverge in the chromosomal phylogeny, we suggest that the CH amplification process is a homoplasy, since heterochromatin usually does not carry functional genes and is unlikely to act in the radiation process, as described for rodents^12,36,37,42,43^.

In total, *C. langguthi* underwent 9 fusions, 2 fissions, 5 pericentric inversions, 1 centromere repositioning, and 1 CH amplification; *C. vivoi* underwent 6 fusions, 1 fission and 2 pericentric inversions; *C. subflavus* underwent 4 fusions, 1 fission and 3 pericentric inversions; *C. scotii* underwent 1 fusion, 4 pericentric inversions and 1 CH amplification (Fig. 7). We suggest that the karyotypic diversity is associated with species radiation. In fact, Di-Nizo et al.^25^ proposed that historical events that occurred in the Pleistocene played an important role in the diversification of *Cerradomys*, in which chromosomal rearrangements in isolated populations may have triggered the speciation events. This process could lead to a rupture in gene flow in a secondary contact caused by the expansion of derived populations with new karyotypic forms^44^.

As previously stated, in Myomorpha rodents, morphological, molecular and chromosomal traits of the group do not evolve at the same rate^12,22^, as in *Oxymycterus* (Sigmodontinae), in which species radiation was not followed by chromosomal diversity, as seven out of the 16 valid species exhibit a conserved karyotype, with the maintenance of 2n with 54 chromosomes and small variation in FNa from 60 to 64^6,26,45^. Another situation is when this mismatch in the rate of evolution of different traits results in cryptic species (two or more distinct species, but with similar morphological traits)^46^, as observed in the genera *Akodon* and *Necromys* (Sigmodontinae), *Lasiopodomys* (Arvicolinae), and *Nannospalax* (Spalacinae)^40,47–49^.

Here, we observed a case in which these three traits coevolved during the speciation process of *Cerradomys*. Similar results were detected in Neotropical rodents of the genus *Neacomys* (Sigmodontinae), in which a gradient of chromosomal and molecular differentiation of four candidate species were reflected in morphological differences that validated these taxa as new species and indicated that speciation was linked to chromosomal variability^21,31,50,51^. Also, a meta-analysis approach compared 41 pairs of rodent sister species and found significant differences in the number of chromosomal traits between sympatric and allopatric species^52^, compatible with a direct role of chromosomal rearrangements in speciation.

## Conclusions

We herein report that the *Cerradomys* genus underwent an extensive karyotypic evolutionary process, with multiple rearrangements that shaped extant karyotypes. Our results show that the chromosomal phylogeny corroborates the phylogenetic relationships proposed by molecular analysis in this genus and indicates that chromosomal rearrangements acted in the speciation process, alongside biogeographic changes in South America that shaped the distribution of extant species.

## Methods

### Samples

**Figure 8:**
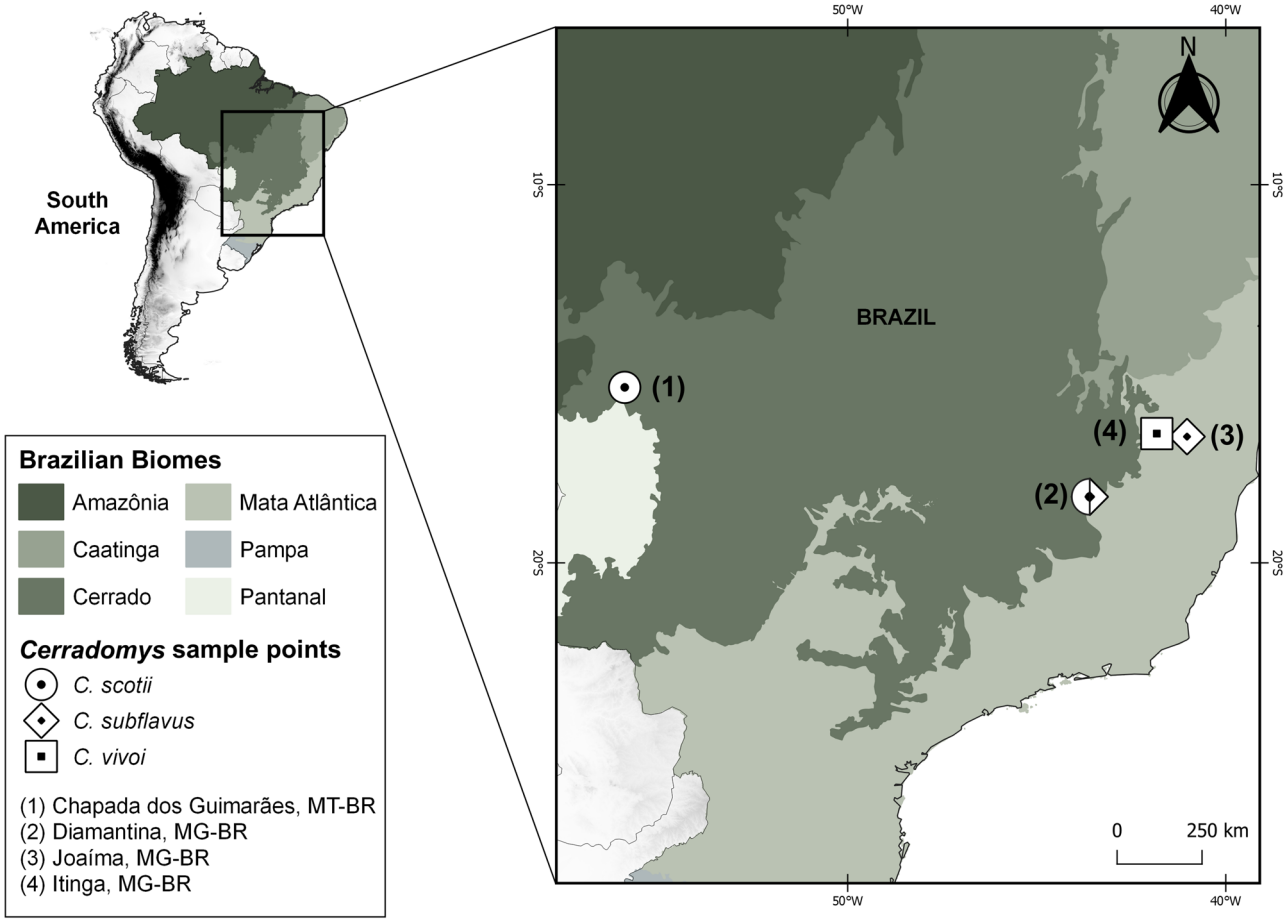
The map shows the study area and sampling points for *Cerradomys* specimens collected in the current study. At each locality, symbols indicate the species that were collected; multiple symbols indicate that more than one species was collected in the same locality. Brazilian biomes are also shown. Brazilian (BR) states are: Mato Grosso (MT) and Minas Gerais (MG).

We studied the karyotypes of wildlife samples of the genus *Cerradomys* (Fig. 8) as follow: three adult samples of *Cerradomys scotii*, of which one male (UFMT4931) and one female (UFMT4932) were from Chapada dos Guimarães, Mato Grosso state, Brazil (15° 21′ 34.1″ S 55° 53′ 53.1″ W), and one female (MN82753) was from Diamantina, Minas Gerais state, Brazil (18° 15′ 08.5″ S 43° 35′ 52.5″ W); three adult samples of *Cerradomys subflavus*, of which one female (MN82759) was from Diamantina, Minas Gerais state, Brazil (18° 15′ 08.5″ S 43° 35′ 52.5″ W), and two males (MN82755, MN82756) from Joaíma, Minas Gerais state, Brazil (16° 39′ 44.2″ S 41° 01′ 29.6″ W); two adult samples of *Cerradomys vivoi*, of which one female (MN82764) and one male (MN82766) were from Itinga, Minas Gerais state, Brazil (16° 34′ 52.3″ S 41° 49′ 27.3″ W). The specimens were deposited at the zoological collections of Museu Nacional, Universidade Federal do Rio de Janeiro (MN), and the Universidade Federal de Mato Grosso (UFMT). Both institutions are in Brazil.

### Cytogenetics 

Metaphase chromosomes were obtained from bone marrow^53^, and were examined by C-Banding^54^, G-Banding^55^ and FISH^56^ techniques. The FISH experiments were performed with 24 whole chromosome painting probes from a female of *Hylaeamys megacephalus* (HME; 2n = 54)^28^, in which 21 peaks correspond to one chromosome each (including the X chromosome), and three peaks corresponded to two pairs of HME chromosomes each (HME (9,10), (13,22) and (16,17)).

### Image capture and analysis 

We used an Olympus BX41 microscope with a CCD 1300QDS digital camera to obtain digital images from G-banded and C-banded karyotypes, which were analyzed using the GenASIs software v. 7.2.7.34276. A Nikon H550S microscope with a DS-Qi1Mc digital camera captured the FISH images, which were analyzed using Nis-Elements software. The karyotypes were organized according to literature^57^. The final images were edited using Adobe Photoshop software v. 22.1.1.

### Map

The map (Fig. 8) was made in software QGIS v. 3.10.7. The shapefiles containing geographic data (country limits) were obtained from DIVA-GIS^58^, available at https://www.diva-gis.org/gdata.

### Syntenic blocks shared among Eumuroida rodents 

An analysis of 58 rodent species hybridized by HME and MMU whole chromosome probes was conducted combining the literature and present study (Additional File 2: Table S2). Using *Necromys lasiurus* karyotypes previously investigated by HME^14^ and MMU probes^13^, syntenic blocks shared among taxa were identified and linked with syntenic blocks that were part of the ancestral Eumuroida karyotype (AEK)^35^.

### Phylogenetic analysis

A list of 56 characters and character states was created by Oliveira da Silva et al.^21^ based on the karyotypes of *Neacomys vossi* (NVO), *N. elieceri* (NEL)^31^, *N. amoenus* (NAM), *N. xingu* (NXI), *N. marajoara* (NMA), *N. paracou* (NPA), *Neacomys* sp. E (NSP-E)^21^, *Hylaeamys megacephalus* (HME), *Cerradomys langguthi* (CLA)^28^, *Thaptomys nigrita* (TNI), *Akodon montensis* (AMO)^29^, *Akodon diauarum* (ADI), *Necromys lasiurus* (NLA)^14^, *Oecomys catherinae* from Pará (OCA-PA) and *O. catherinae* from Rio de Janeiro (OCA-RJ)^30^. Here, we used the same list and integrated 14 new characters (a total of 70) by including the karyotypes of *Oecomys auyantepui* (OAU)^33^, *O. paricola* cytotype A (OPA-A), *O. paricola* cytotype B (OPA-B), *O. paricola* cytotype C (OPA-C)^32^, *Oxymycterus amazonicus* (OAM), *Blarinomys breviceps* (BBR)^6^, *Rhipidomys mastacalis* (RMA), *R. emiliae* (REM)^34^, *Cerradomys scotii* (CSC), *C. subflavus* (CSU), and *C. vivoi* (CVI) (present work). As a result, the present analysis increased the number of taxa (from 14 to 25), genera (from 6 to 9) and tribes due to the inclusion of Thomasomyini (RMA, REM) taxa.

The chosen non-additive multi-state characters were chromosomal morphology, number and syntenic blocks based on hybridization with *Hylaeamys megacephalus* (HME) whole chromosome probes^28^ (Additional File 3: Table S3; Additional File 4). The new list of characters and character states was converted into a non-additive (unordered) multi-state character matrix (Additional File 5: Table S4) on Mesquite program version 3.70^59^.

The Maximum Parsimony (MP) phylogenetic analysis was made using T.N.T. (Tree analysis using New Technology) program version 1.6^60^. Branch support values were calculated with 1000 bootstrap replicates. The exhaustive search was made using Tree Bisection Reconnection (TBR). We used RMA as outgroup, a taxon from the Thomasomyini tribe which, alongside with Akodontini and Oryzomyini, belong to the Sigmodontinae subfamily^61^. The tree was displayed and edited in Figtree program version 1.4.2 (http://tree.bio.ed.ac.uk/software/figtree/). Gaps were read as “missing data”.

### Ethics approval and consent to participate

The specimens were captured using live traps (Sherman and Tomahawk) and pitfall traps^62^ and kept stress-free with full access to food and water until their necessary euthanasia was performed in accordance with animal welfare guidelines established by Brazilian resolution CFMV n.1000/2012. The euthanasia was carried out in accordance with animal welfare guidelines established by the Animal Ethics Committee (Comitê de Ética Animal) from Universidade Federal do Pará (Permit 68-2015), which also approved all experimental protocols of this research. The captures were authorized by the Brazilian Environment Department under license (IBAMA 02,047.000384/2007-34). JCP has a permanent field permit (number 13248) from “Instituto Chico Mendes de Conservação da Biodiversidade”. The Cytogenetics Laboratory from UFPa has a special permit number 19/2003 from the Ministry of Environment for samples transport and 52/2003 for using the samples for research. All methods are reported in accordance with ARRIVE guidelines (https://arriveguidelines.org/).

### Supplementary Information

Supplementary Information 1.

Supplementary Information 2.

Supplementary Information 3.

Supplementary Information 4.

Supplementary Information 5.

## Data Availability

All results reported in this article are found in the main text and in the supplementary files.

## Abbreviations

Cytb: Cytochrome b
COI: Cytochrome C Oxidase Subunit I
BI: Bayesian Inference
ML: Maximum Likelihood
MP: Maximum Parsimony
FISH: Fluorescence in situ hybridization
2n: Diploid number
FNa: Autosomal fundamental number
CH: Constitutive heterochromatin
HSA: Human whole chromosome probes
MMU: *Mus musculus*
HME: *Hylaeamys megacephalus*
NVO: *Neacomys vossi*
NEL: *Neacomys elieceri*
NAM: *Neacomys amoenus*
NXI: *Neacomys xingu*
NMA: *Neacomys marajoara*
NPA: *Neacomys paracou*
NSP-E: *Neacomys* Sp. E
CLA: *Cerradomys langguthi*
CSC: *Cerradomys scotii*
CSU: *Cerradomys subflavus*
CVI: *Cerradomys vivoi*
OCA-PA: *Oecomys catherinae* From Pará
OCA-RJ: *Oecomys catherinae* From Rio de Janeiro
OAU: *Oecomys auyantepui*
OPA-A: *Oecomys paricola* Cytotype A
OPA-B: *Oecomys paricola* Cytotype B
OPA-C: *Oecomys paricola* Cytotype C
OAM: *Oxymycterus amazonicus*
BBR: *Blarinomys breviceps*
RMA: *Rhipidomys mastacalis*
REM: *Rhipidomys emiliae*
TNI: *Thaptomys nigrita*
AMO: *Akodon montensis*
ADI: *Akodon diauarum*.
NLA: *Necromys lasiurus*
T.N.T.: Tree Analyses Using New Technologies
TBR: Tree Bisection Reconnection
