# Supplementary material for: Chromosomal rearrangements played an important role in the speciation of rice rats of genus Cerradomys (Rodentia, Sigmodontinae, Oryzomyini)
Source: Sci Rep. 2024 Jan 4;14:545. doi: 10.1038/s41598-023-50861-3 (PMC10766967; doi:10.1038/s41598-023-50861-3)
Supplement: Supplementary file 3 — Supplementary Information 3. [file 41598_2023_50861_MOESM3_ESM.docx]

Table S3 Non-additive multi-state character list. Character descriptions are detailed in Additional File 4. Characters 1-56 were previously described [21] and characters 57-70 are from the present study

| **Character** | | **Non-additive multi-state characters** | | | |
| --- | --- | --- | --- | --- | --- |
|  |  | **0** | **1** | **2** | **3** |
| **1** | HME 1 chromosome | 1(a+b+c) | 1a+1(b+c) | 1a+1b+1c |  |
| **2** | HME 2 chromosome | 2(a+b+c) | 2a+2(b+c) | 2a+2b+2c |  |
| **3** | HME 3 chromosome | 3(a+b) | 3a+3b |  |  |
| **4** | HME 4 chromosome | 4(a+b+c) | 4(a+c)+4b | 4a+4b+4c |  |
| **5** | HME 5 chromosome | 5(a+b+c+d) | 5(a+b)+5(c+d) | 5(a+b)+5c+5d | 5a+5b+5c+5d |
| **6** | HME 6 chromosome | 6(a+b+c) | 6a+6(b+c) | 6a+6b+6c |  |
| **7** | HME 7 chromosome | 7(a+b) | 7a+7b |  |  |
| **8** | HME 8 chromosome | 8(a+b) | 8a+8b |  |  |
| **9** | HME 11 chromosome | 11(a+b+c+d) | 11(a+b)+11(c+d) | 11a+11b+11(c+d) | 11a+11b+11c+11d |
| **10** | HME 14 chromosome | 14(a+b) | 14a+14b |  |  |
| **11** | HME 18 chromosome | 18(a+b+c) | 18a+18(b+c) |  |  |
| **12** | HME 19 chromosome | 19(a+b) | 19a+19b | 19a+19b1+19b2 |  |
| **13** | HME 22 chromosome | 22(a+b) | 22a+22b |  |  |
| **14** | HME 23 chromosome | 23(a+b) | 23a+23b |  |  |
| **15** | HME 24 chromosome | Metacentric | Acrocentric |  |  |
| **16** | HME 25 chromosome (I) | 25(a+b) | 25a+25b |  |  |
| **17** | HME 25 chromosome (II) | Metacentric | Acrocentric |  |  |
| **18** | HME 26 chromosome | Metacentric | Acrocentric |  |  |
| **19** | Chromosomal association HME 1/12 | Absent | Present |  |  |
| **20** | Chromosomal association HME 1/20/(13,22) | Absent | Present |  |  |
| **21** | Chromosomal association HME 1/12/2/18/4/(16,17)/11 | Absent | Present |  |  |
| **22** | Chromosomal association HME 18/2 | Absent | Present |  |  |
| **23** | Chromosomal association HME 2/23 | Absent | Present |  |  |
| **24** | Chromosomal association HME 3/25 | Absent | Present |  |  |
| **25** | Chromosomal association HME 3/25/18 | Absent | Present |  |  |
| **26** | Chromosomal association HME 4/18 | Absent | Present |  |  |
| **27** | Chromosomal association HME 4/11/(16,17) | Absent | Present |  |  |
| **28** | Chromosomal association HME 8/(13,22)/5/(16,17) | Absent | Present |  |  |
| **29** | Chromosomal association HME 5/(9,10) | Absent | Present |  |  |
| **30** | Chromosomal association HME 5/(13,22) | Absent | Present |  |  |
| **31** | Chromosomal association HME 5/18 | Absent | Present |  |  |
| **32** | Chromosomal association HME 5/11 | Absent | Present |  |  |
| **33** | Chromosomal association HME (9,10)/14/5 | Absent | Present |  |  |
| **34** | Chromosomal association HME 6/21 | Absent | Present |  |  |
| **35** | Chromosomal association HME 6a/21 | Absent | Present |  |  |
| **36** | Chromosomal association HME 7/(9,10) | 7/(9,10) | 7b/(9,10) | (9,10)/7b/(9,10) |  |
| **37** | Chromosomal association HME (9,10)/15 | Absent | Present |  |  |
| **38** | Chromosomal association HME (13,22)/11 | Absent | Present |  |  |
| **39** | Chromosomal association HME 11/(16,17) | Absent | Present |  |  |
| **40** | Chromosomal association HME 12/(16,17) | Absent | Present |  |  |
| **41** | Chromosomal association HME 20/(13,22) | Absent | Present |  |  |
| **42** | Chromosomal association HME 20/(13,22)/4 | Absent | Present |  |  |
| **43** | Chromosomal association HME 14/19 | Absent | Present |  |  |
| **44** | Chromosomal association HME 19a/14/19b | Absent | Present |  |  |
| **45** | Chromosomal association HME 19a/14/19b1+19b2 | Absent | Present |  |  |
| **46** | Chromosomal association 19a/14 | Absent | Present |  |  |
| **47** | Chromosomal association HME 19/14/23 | Absent | Present |  |  |
| **48** | Chromosomal association HME 19/7 | Absent | Present |  |  |
| **49** | Chromosomal association HME 23/19/11 | Absent | Present |  |  |
| **50** | Chromosomal association HME 3/25/6/21 | Absent | Present |  |  |
| **51** | Chromosomal association HME (13.22)/26 | Absent | Present |  |  |
| **52** | Chromosomal association HME (13,22)/21 | Absent | Present |  |  |
| **53** | Chromosomal association HME 26/11 | Absent | Present |  |  |
| **54** | Chromosomal association HME 5/19/14/19/5 | Absent | Present |  |  |
| **55** | Chromosomal association HME 18/25 | Absent | Present |  |  |
| **56** | Chromosomal association HME 24/5/(13,22)/11 | Absent | Present |  |  |
| **57** | Chromosomal association HME 1/8 | Absent | Present |  |  |
| **58** | Chromosomal association HME 1/14 | Absent | Present |  |  |
| **59** | Chromosomal association HME 1/20 | Absent | Present |  |  |
| **60** | Chromosomal association HME 19/7/(9,10) | Absent | Present |  |  |
| **61** | Chromosomal association HME 3/19/7/(9,10) | Absent | Present |  |  |
| **62** | Chromosomal association HME 4b/19 | Absent | Present |  |  |
| **63** | Chromosomal association HME 5/15 | Absent | Present |  |  |
| **64** | Chromosomal association HME (13,22)/15/11 | Absent | Present |  |  |
| **65** | Chromosomal association HME 6/21/8 | Absent | Present |  |  |
| **66** | Chromosomal association HME 12/(9,10) | Absent | Present |  |  |
| **67** | Chromosomal association HME 15/12/(9,10) | Absent | Present |  |  |
| **68** | Chromosomal association HME 18/20/(13,22) | Absent | Present |  |  |
| **69** | Chromosomal association HME (13,22)/4 | Absent | Present |  |  |
| **70** | Chromosomal association HME (13,22)/26/25/3 | Absent | Present |  |  |
